# Clinical performance of a dual-target SARS CoV-2 antibody assay using sera from Ghana

**DOI:** 10.1186/s12879-025-12012-z

**Published:** 2025-11-12

**Authors:** Prince Jonathan Pappoe-Ashong, Julius Abraham A. Mingle, Derrick Tetteh, Martha S. Dsane-Lamptey, Joseph A. Oliver-Commey, Peter Puplampu, Christian Jassoy

**Affiliations:** 1 Institute of Medical Microbiology and Virology, University Clinics and Medical Faculty, University of Leipzig, Leipzig, Germany; 2 Department of Medical Microbiology, University of Ghana Medical School, Accra, Ghana; 3 Department of Medicine, University of Ghana Medical School, Accra, Ghana; 4 Ghana Infectious Disease Centre, Ghana Health Service, Accra, Ghana; 5 Ga East Municipal Hospital, Ghana Health Service, Accra, Ghana

**Keywords:** Serology, Performance characteristics, Receptor binding domain (RBD), Nucleocapsid protein (NP)

## Abstract

**Background:**

During the pandemic, numerous serological tests for severe acute respiratory syndrome coronavirus 2 (SARS CoV-2) have been developed. However, limited data exist on the clinical performance of these immunoassays with sera from Africa. The aim of this study was to determine the clinical performance characteristics of a lateral flow IgG antibody test using serum and plasma samples from Ghana.

**Method:**

We assessed the performance of the COVID-19 Sero-NP/RBD test, a dual-target lateral flow assay designed to simultaneously detect IgG antibodies against the SARS-CoV-2 nucleoprotein (NP) and spike receptor-binding domain (RBD). Diagnostic sensitivity and specificity were determined using pre-pandemic plasma samples (*N* = 300) and samples from recovered COVID-19 patients with varying symptom severity (*N* = 308). Test performance was further analyzed in relation to antibody concentration.

**Results:**

The combined detection of anti-RBD and anti-NP antibodies yielded a diagnostic sensitivity of 96.1% (95% CI: 94.1–98.4) and a specificity of 99.0% (95% CI: 97.1–99.8). Combined antigen detection significantly outperformed individual antigen targets (*p* < 0.001), with RBD-targeted responses demonstrating higher sensitivity than NP alone. Sensitivity declined with lower antibody titres, decreasing to 86.0% for samples with ≤ 25 BAU/mL and to 57.0% for those with ≤ 7.5 BAU/mL when either antigen was used alone.

**Conclusion:**

The COVID-19 Sero-NP/RBD lateral flow test demonstrated high accuracy in detecting past SARS CoV-2 infection in sera from Ghana. Dual-antigen targeting enhanced diagnostic sensitivity, particularly in individuals with low antibody titres. This assay may serve as a practical tool for assessing individual serostatus and monitoring population-level seroprevalence in Ghana and similar African settings.

## Introduction

Since the beginning of the SARS CoV-2 pandemic in 2019, numerous immunoassays have been developed to detect antibodies against SARS CoV-2. Immunoassays are analytical methods that use antigen-antibody interactions to detect or quantify various substances including endogenous proteins, toxins, or proteins derived from infectious agents. The latter are also referred to as antigens. These assays serve a wide array of purposes, including clinical diagnostics and vaccine evaluation, epidemiological surveillance and therapeutic monitoring. Immunoassays may be quantitative, measuring the concentration of a substance, or qualitative, indicating whether a particular substance is present or absent. Commonly used immunoassay formats include enzyme-linked immunosorbent assays (ELISAs), chemiluminescent immunoassays (CLIAs), and lateral flow assays (LFAs). These differ in technical design and clinical application. LFAs are immunochromatographic tests that typically yield results within 15–30 min and are especially valuable in point-of-care and resource-limited settings. In the context of SARS CoV-2, LFAs can be designed as antigen tests, which detect viral proteins to identify current infection, or as antibody tests, which detect host immunoglobulin (IgM, IgG, or total Ig) responses to viral antigens, providing evidence of prior exposure or vaccination. Most antibody LFAs for SARS CoV-2 target the nucleocapsid protein (NP), the spike receptor-binding domain (RBD), or both [1, 2]. To date, approximately 174 antibody-based LFAs or rapid diagnostic tests have been developed for SARS CoV-2 [3]. In Africa, many assays used in seroprevalence studies are antibody LFAs or rapid diagnostic tests (RDTs), reflecting their wide adoption and accessibility across the continent [4].

Immunoassays are typically certified as medical devices using clinical samples derived from populations in the region of manufacture—most often Europe, North America, or Asia. However, such certification does not necessarily guarantee optimal performance across all global populations. In sub-Saharan Africa, where infectious diseases such as malaria, HIV, and dengue fever are endemic, individuals may present with distinct serological profiles that differ significantly from those in temperate regions. These differences can impact assay specificity and overall diagnostic accuracy [2, 5]. Therefore, regional performance evaluations are critical — not for regulatory approval, but to confirm the reliability and suitability of immunoassays in specific local populations. For example, a validation study of commercial SARS CoV-2 immunoassays conducted in a Nigerian population revealed variations in diagnostic performance, highlighting the importance of context-specific validation [2]. Across individual studies, reported diagnostic sensitivities and specificities for antibody tests have ranged widely from 0% to 100% [6–12].

In many African countries, rapid tests are more practical than laboratory tests due to limited medical laboratory capacity. One such rapid test for SARS CoV-2 is the COVID-19 Sero NP/RBD Lateral Flow Test. The test is designed to simultaneously detect and distinguish SARS CoV-2 NP and RBD IgG antibodies.

The aim of this study was to investigate the diagnostic sensitivity and specificity of this test using serum and plasma samples from Ghana. Furthermore, the performance of individual antibody markers (anti-NP or anti-RBD) was to be compared to the combined (anti-NP and or anti-RBD) antibody format.

## Methodology

### Study design

This was a diagnostic test evaluation study to determine the clinical sensitivity and specificity of the COVID-19 Sero NP/RBD lateral flow test using sera from Ghana.

### Setting and samples

Plasma and serum (*n* = 308) from SARS CoV-2-infected symptomatic individuals aged 10 to 89 years (median age 40 years) were used for the investigation. Sample size was based on feasibility and consistent with STARD 2015 recommendations [13]. Further, post hoc power analysis indicated that a total sample size of approximately 264 (88 per group) would be required to detect a moderate effect size (Cohen’s w = 0.3) with 80% power at α = 0.05. Although the moderate disease group included 59 participants — slightly below this per-group estimate — the overall sample size (*n* = 308) exceeded the requirement, and subgroup comparisons still yielded statistically significant differences in sensitivity across severity groups.

The samples were collected at Korle-bu Teaching Hospital, Accra, Ghana, and the Ghana Infectious Disease Centre, Ghana Health Service, between May 2020 and October 2021. Samples were collected between 5 and 178 days (median 22 days) after RT-PCR, mostly at follow-up after SARS CoV-2 infection. To evaluate the specificity of the test, pre-pandemic plasma (*N* = 300) from individuals aged 2.1 to 64 years (median age 26 years) from 2008 to 2010 were used. All these samples were collected primarily for clinical viral diagnostics and screening purposes. Of these, 54% (*N* = 162) of the samples were HBsAg positive.

### Combined NP/RBD lateral flow evaluation

The Coris BioConcept COVID-19 Sero NP/RBD™, manufactured in Belgium, is a dual-target lateral flow assay designed to simultaneously detect IgG antibodies against the SARS CoV-2 NP and RBD. The test contains the two SARS CoV-2 virus antigens NP and RBD in a test cassette. The samples were tested according to the manufacturer’s protocol. This involved adding 30 µl of serum to the test cassette, followed by 4 drops of buffer after 10 s. After 15 min, the results were read, photographed and documented. Band intensities were graded on a 0–9 scale and recorded separately for NP, RBD, and control bands. An intensity of 0 was considered negative and 1–9 positive, although very weak bands not consistently identified by multiple readers were classified as negative. According to the manufacturer’s instructions for use (tested with sera from patients infected with SARS CoV-2), the diagnostic sensitivity of the test was as follows: NP = 95.2%, RBD = 91.9% and combined (i.e., if either or both bands were positive) NP/RBD 98.4%. The specificity was for NP = 98.5%; RBD = 100.0% and combined NP/RBD 98.5% (i.e., if both bands were negative).

### Quantification of antibody RBD and NP titre

Antibody concentrations of RBD (*N* = 155) and NP (*N* = 124) were determined using internal NP and RBD ELISA tests.

To determine the RBD and NP titres of each serum and plasma sample, high-binding 96-well microtiter plates (Greiner BioOne) were coated with 2 µg/ml NP-MBP or with 1 µg/ml RBD in phosphate-buffered saline (PBS), pH 7.4. The microtiter plates were incubated overnight at 4 °C. All subsequent steps were performed at room temperature. The plates were washed with deionised water and washing buffer (PBS, 0.05% Tween-20) and blocked with blocking solution (PBS with 0.05% Tween-20 and 5% milk powder) for 20 min. The standards that were used are the first WHO International Standard for anti-SARS CoV-2 immunoglobulin (NIBSC, 20/136 (for NP IgG quantification) and serum with 250 BAU/ml calibrated with Abbott IgG assay (for RBD IgG quantification). Plasma or serum samples were diluted 1:100 in blocking solution and incubated for 60 min. All microtiter plates were washed and a goat anti-human IgG conjugated with HRP (Art. No. 109-036-098, Jackson ImmunoResearch) diluted 1:40,000 for the NP-based test and 1:20,000 for the RBD was added for 60 min. The microtiter plates were washed and 3,3′,5,5′-tetramethylbenzidine (SeramunBlue slow 2/85, Seramun Diagnostica GmbH) was used as substrate. The enzymatic reaction was stopped after 15 min by adding 1 N sulphuric acid. The optical density (OD) was measured using a photometer at a wavelength of 450 nm and a reference wavelength of 570 nm. Samples that showed higher antibody titre than the threshold OD measurement were further diluted and re-analysed.

### Statistical analysis

All data were recorded in Microsoft Excel, LTSC professional plus 2021. Statistical analyses were performed using GraphPad Prism 8 on Windows 64-bit. Confidence intervals were set at 95% and were calculated using the MedCalc test ‘Test for one proportion’. The days after blood drawing in groups with different courses of disease were compared using the Kruskall-Wallis test (https://www.socscistatistics.com/tests/kruskal/default.aspx). The clinical sensitivity and specificity, predictive values and accuracy of assay was calculated using MedCalc ‘Diagnostic test evaluation calculator’ (https://www.medcalc.org/).

## Results

### Demographic and clinical characteristics of study samples

The majority of the total 308 samples from SARS CoV-2 infected symptomatic individuals, were from males (*n* = 172, 55.8%). In this group, age 30–39 had the highest frequency (*n* = 90, 29.2%). The sera were from patients with mild (*n* = 129, 42%), moderate (*n* = 56, 18%) and severe (*n* = 123, 40%) disease. The majority of the 300 pre-COVID-19 samples were from females (*n* = 177, 59%) and from the age groups 20–29 years (*n* = 181, 60.3%) (Table 1). The median number of days from symptom onset to blood collection was 22 days for both mild and severe disease, and 23 days for moderate disease. However, there was no significant difference in days after sampling for all disease statuses (Table 2).

**Table 1:** Demographic and clinical characteristics of study participants

Characteristics				
***Sex***	COVID − 19		Pre COVID-19	
	**Number of sera**	**% of total**	**Number of sera**	**% of total**
Male	172	55.8	123	41.0
Female	136	44.2	177	59.0
Total	308	100	300	100
***Age (years)***	COVID − 19		Pre COVID-19	
	**Number of sera**	**% of total**	**Number of sera**	**% of total**
0–9	-	-	2	0.6
10–19	7	2.3	6	2.0
20–29	46	14.9	181	60.3
30–39	90	29.2	70	23.3
40–49	54	17.5	29	9.7
50–59	42	13.6	9	3.0
≥ 60	69	22.4	3	1.0
Total	308	100	300	100
**COVID-19 Disease status**	COVID − 19			
	**Number of sera**	**% of total**	**-**	**-**
Mild	126	40.9	-	-
Moderate	59	19.2	-	-
Severe	123	39.9	-	-
Total	308	100	-	-

**Table 2:** Overall clinical performance characteristics of the assay

COVID-19 Disease status	Period at point sample collection post illness (days)			Clinical sensitivity (%) (95% Confidence interval (CI))			Clinical specificity (%) (95% Confidence interval (CI))		
	Range (Mean)	Median	*p*-value	RBD	NP	Overall^b^	RBD	NP	Overall^b^
Overall COVID-19 (*n* = 308)	5–178 (30.12)	22	-	88.6^1^ (84.55–91.96)	83.8^1^ (79.16–87.70)	96^1^ (94.11–98.43)			
Mild COVID-19 (*n* = 126)	5–148 (32.2)	22	0.1158^a^	75* (66.93–82.63)	67** (57.72–74.81)	93 (86.87–96.68)	-	-	-
Moderate COVID-19 (*n* = 59)	8–149 (37.6)	23		93* (83.54–98.12)	88** (77.07–95.09)	98.31 (90.91–99.96)	-	-	-
Severe COVID-19 (*n* = 123)	5–178 (24.27)	22		100^#^ (97.05–100.00)	100^#^ (97.05–100.00)	100 (97.05–100.00)	-	-	-
Pre-COVID-19 (*n* = 300)	-	-	-	-	-	-	100 (98.78–100.00)	99 (97.11–99.79)	99 (97.11–99.79)

^a^The difference in the number of days post-illness was not statistically significant among all three disease statuses (*p* > 0.05). Significant differences (*p* < 0.05) in diagnostic/clinical sensitivity: *p value = 0.0039 (mild vs. moderate), **p value = 0.00261 (mild vs. moderate), ^#^p value < 0.0001 (mild vs. severe). ^b^Overall means detected by the NP and/or RBD marker. ^1^Significant difference in diagnostic sensitivity between RBD alone and NP alone vs. the combination of NP and/or RBD markers, *p* < 0.001

### Clinical performance characteristics of the test

The clinical specificity of the COVID-19 Sero NP/RBD rapid test was 99% (95% CI, 97.11–99.79) for combined markers (RBD or NP antibody). The specificity with the RBD antibody marker alone was 100% (95% CI, 98.78–100.00) and with the NP antibody marker 99% (95% CI, 97.11–99.79).

The clinical sensitivity was 96% (95% CI, 94.11–98.43) for the combined RBD and NP antibody marker. The clinical sensitivity with the RBD antibodies was 88.6% (95% CI, 84.55–91.96) and with the NP antibodies 83.8% (95% CI, 79.16–87.70). Subgroup analysis of the performance of the assay with samples from different disease courses showed a diagnostic sensitivity for RBD and or NP antibody of 93% (95% CI, 86.87–96.68) for the mild, 98.1% (95% CI, 90.91–99.96) for the moderate and 100% (95% CI, 90.91–99.96), for the severe disease. The individual antibody markers of RBD only and NP only was 75% (95% CI, 66.93–82.63) and 67% (95% CI, 57.72–74.81) for mild, 93% (95% CI, 83.54–98.12) and 88% (95% CI, 77.09–95.09) for moderate, and both 100% (95% CI, 97.05–100) for severe disease states. Thus, the clinical sensitivity of the individual antibody markers RBD and NP was lower among samples from the mild disease course than the other two disease statuses. Also, the RBD antibody marker was relatively more sensitive than the NP antibody marker in samples from the mild and moderate disease courses but this was not statistically significant (Table 2).

### Predictive value of the RBD and NP IgG bands

To calculate the predictive value of the assay, we used a pre-test probability of 51% based on the seroprevalence of SARS CoV-2 recorded in Accra, Ghana, between August and October 2020 (own unpublished data (manuscript in preparation)).

Overall, in this epidemiological scenario the assay showed good predictive values and accuracy. The positive predictive values (PPV) of the combined NP/RBD, NP and RBD markers were 99.02% (95% CI: 97.03% − 99.68%), 100% (95% CI: 98.66% − 100%) and 98.87% (95% CI: 96.58% − 99.63%), which was not statistically different. The negative predictive values (NPV) of the combined NP/RBD, NP and RBD markers alone were 96.7% (95% CI: 94.09% − 98.18%) and 89.42% (95% CI: 86.09% − 92.03%) (RBD and NP), which shows a significantly better NPV of the combined NP/RBD marker. The accuracy of the combined NP/RBD, NP and RBD markers were 97.85% (95% CI: 96.36% − 98.85%), 94.20% (95% CI: 92.04% − 95.93%) and 91.23% (95% CI: 88.69% − 93.36%), respectively, underlining that the use of both markers increases the precision of the assay compared with single markers alone (Table 3).

**Table 3:** Predictive values of the assay

Antibody marker(s)	Number of samples	Positive predictive value (95% CI)	Negative predictive value (95% CI)	Accuracy ( 95% CI)
NP/RBD	608	99.02% (97.03% − 99.68%)	96.70% (94.09% − 98.18%)	97.85% (96.36% − 98.85%)
RBD	608	100.00% (98.66% − 100.00%)	89.42% (86.09% − 92.03%)	94.20% (92.04% − 95.93%)
NP	608	98.87% (96.58% − 99.63%)	85.42% (81.97% − 88.31%)	91.23% (88.69% − 93.36%)

*Prevalence used in these calculations is 51% (on the basis of the seroprevalence of SARS CoV-2 recorded in Ghana between August and October 2020 (own unpublished data))

### Comparison of the clinical performance of the assay between low and high titre samples

**Fig. 1:**
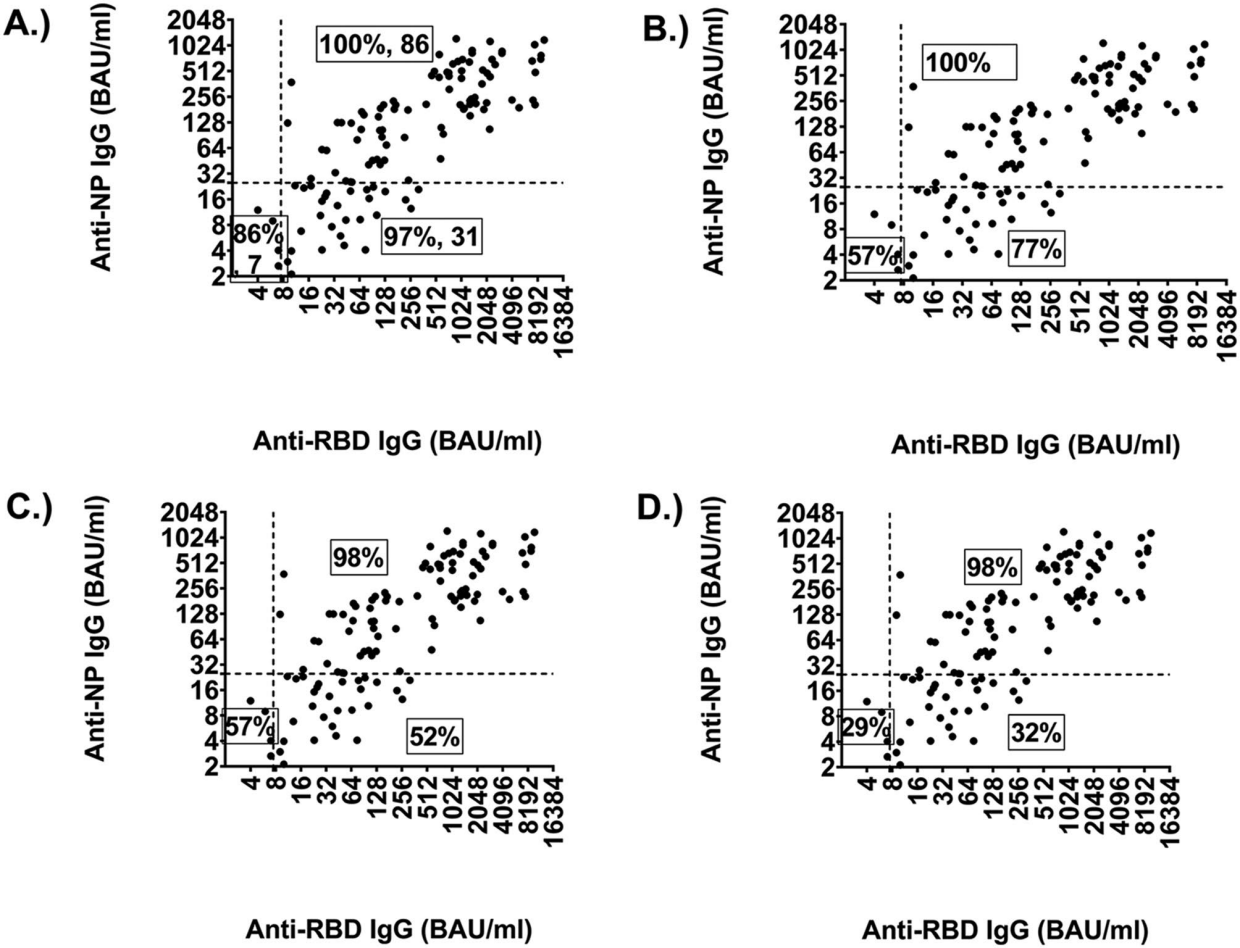
Diagnostic sensitivities of the assay with sera grouped according to the anti-RBD- and NP-IgG concentrations using one or two bands as readout. Dashed lines indicate the lower limits of detection (LOD) of the assay. The upper right quadrant shows the sera with anti-RBD- and NP-IgG concentrations above the LODs (number of sera (N) = 86), the lower right quadrant shows sera with anti-RBD IgG levels above the LOD and anti-NP IgG concentrations below the LOD (*N* = 31). The lower left quadrant contains sera with IgG concentrations below the LOD for RBD and NP antibodies (N) = 7). Diagnostic sensitivity for (**A**) NP or RBD band or both bands; (**B**) RBD band only; (**C**) NP band alone; (**D**) NP plus RBD band

We also compared the diagnostic sensitivity of the test when we evaluated one or both bands in groups with low and higher antibody concentrations. To do this, we arbitrarily divided the sera into four groups with anti-RBD IgG concentrations below and above 7.5 BAU/ml and anti-NP IgG below and above 25 BAU/ml. Test evaluation using one or both bands showed similar sensitivity of 98–100% in the group with higher antibody concentrations (Fig. 1, upper right quadrant). In comparison, sensitivity differed significantly in the groups with low NP-specific IgG concentrations. 97% of samples in the group with low NP values but higher RBD-specific IgG showed either an RBD and or NP IgG band. 77% of sera showed only an RBD IgG band, 52% of sera showed an NP IgG band, and 32% of sera showed both bands (Fig. 1 lower right quadrant). This is similar to the sera in the lower left quadrant (although the number of sera is small) with RBD or NP bands giving a sensitivity of 86% (Fig. 1A) compared to 57% with RBD IgG alone (Fig. 1B) or NP IgG alone (Fig. 1C). Therefore, this indicates that the diagnostic sensitivity of each marker is significantly affected by the antibody concentration in the serum or plasma being analysed.

## Discussion

Several commercially developed LFTs detect IgG or IgM antibodies against the viral nucleocapsid protein (NP) or portions of the spike glycoprotein (S1 or S2) [9, 12, 14]. Fewer assays incorporate both NP and the receptor-binding domain (RBD) within a single cartridge [15]. This dual-target strategy accommodates inter-individual variability in immune responses and can enhance diagnostic sensitivity without compromising specificity. In this study, we evaluated a lateral flow assay capable of simultaneously detecting and differentiating anti-NP and anti-RBD antibodies. Interpreting the test as positive if either target was detected improved sensitivity and overall diagnostic accuracy compared with either antigen alone.

The assay demonstrated high diagnostic sensitivity (96%) and specificity (99%), comparable to other commercial SARS CoV-2 antibody tests targeting NP and/or RBD IgG [7, 16–18]. Predictive values were similarly strong, supporting potential use in both clinical diagnostics and population-level serosurveillance. However, RBD consistently showed higher sensitivity than NP, in line with prior reports [16, 19–21]. This difference may partly reflect NP assay design. Cross-reactivity is a particular concern for NP-based assays, as pre-existing immunity to seasonal coronaviruses can generate false positives. To minimize this, NP assays are calibrated to require higher antibody concentrations. For example, one validation study stated a substantially higher NP cut-off (9787 AU/mL) compared with Spike (675 AU/mL) or RBD (2396 AU/mL), yet al.l achieved 99% specificity [22], suggesting that stronger NP signals are needed to reach equivalent specificity [23]. Furthermore, one BAU of anti-NP IgG corresponds to ~ 3.5 times more IgG mass than one BAU of anti-RBD IgG [24]. Consequently, a 25 BAU anti-NP IgG threshold corresponds to approximately ten times the IgG content of 7.5 BAU anti-RBD IgG, which further explains the observed differences in sensitivity.

Sensitivity was highest in moderate and severe cases, likely reflecting higher antibody titres in these groups, consistent with evidence linking severe COVID-19 to stronger humoral responses [21, 25]. Incorporating both antigens appears to compensate for variability in immune targeting, enhancing detection in mild disease and at later stages post-infection. These differences in sensitivity prompted us to examine the role of timing of sampling and antibody concentration.

The interval between symptom onset and serum sample collection is an important determinant of SARS CoV-2 antibody assay sensitivity. In this study, samples were collected a median of 22 days post-symptom onset (mean of 30.1 days). The mean exceeding the median indicates a subset of substantially delayed period between time of infection and sampling, likely due to home isolation [26], or late clinic attendance. However, there was no statistically significant timing differences between severity groups (*p* = 0.1158). Despite this, sensitivity varied markedly by severity: lowest in mild cases (RBD 75%, NP 67%) and highest in severe cases (100% for both antigens). This reduced sensitivity in mild disease likely reflects transient or lower-magnitude antibody responses due to lower viral loads and limited immune stimulation. These findings align with previous reports that mild or asymptomatic infections often induce weaker, shorter-lived humoral responses, which can impair assay performance, particularly as time from infection increases [27, 28]. Nonetheless, protective immune responses can occur without symptoms, as seen with vaccines and asymptomatic infections such as hepatitis A, measles, mumps, influenza, and SARS CoV-2, showing that symptom presence is not necessary for robust immunity. Building on this, we next assessed the direct impact of antibody concentration.

Antibody concentration had a marked effect on assay performance. Using arbitrarily defined limits (based on our own dilution series (RBD >7.5 BAU/mL, NP >25 BAU/mL)), high-titre samples resulted in ≥ 98% sensitivity for both antigens, whereas low-titre samples led to substantially reduced sensitivity (RBD 41%, NP 43%) (Fig. 1). The reduced sensitivity observed aligns with previous longitudinal studies showing declines six months post-infection as antibody titres wane [16]. Notably, combining NP and RBD targets improved detection in low-titre samples, confirming earlier reports that dual-antigen assays outperform single-antigen formats [16, 17]. To understand how these findings translate into practice, we evaluated predictive values under different prevalence assumptions.

Predictive value modelling, using an internal first-wave seroprevalence estimate of 51% for Accra, Ghana, showed that PPV, NPV, and accuracy varied with population prevalence. At low prevalence (5–25%), PPV declined while NPV and accuracy improved; at very high prevalence (> 90%), PPV increased modestly but NPV decreased slightly. These shifts highlight the importance of interpreting assay results in their epidemiological context. In high-prevalence settings, the manufacturer’s recommendation to consider any positive band as evidence of prior infection or vaccination is appropriate. In low-prevalence settings, an isolated NP-positive result may be best considered indeterminate, warranting confirmatory testing. These findings also raise the question of assay specificity in populations with high burdens of other infections.

Specificity was excellent (100% for RBD, 99% for NP). Three pre-pandemic serum samples, each testing positive for hepatitis B surface antigen (HBsAg), produced NP-positive results. This suggests possible non-specific binding. A limitation of this study is that we could not further investigate the potential influence of hepatitis B on cross-reactive antibody responses. Cross-reactivity in samples from individuals with malaria, HIV, or HBV infection has been reported in evaluations of other commercial assays [19, 29, 30]. To further address potential cross-reactivity, we recommend targeted validation using panels tested for multiple HBV markers [31, 32], as well as for malaria, HIV, and other locally prevalent pathogens [33, 34], to more robustly assess assay specificity in endemic settings.

Finally, emerging SARS CoV-2 variants present an additional challenge [35]. Variants such as Omicron carry mutations in the spike protein that can reduce the sensitivity of assays targeting antibodies against the viral RBD [36, 37]. The NP antigen is relatively conserved, and its inclusion may help mitigate variant-induced performance loss. Therefore, the combined NP/RBD format improves robustness in situations with high or unknown variant prevalence.

## Conclusion

The COVID-19 Sero NP/RBD rapid test demonstrated high diagnostic accuracy and proved effective for detecting SARS-CoV-2 antibodies following infection. The dual-target design, incorporating IgG against both NP and RBD, enhanced diagnostic sensitivity compared to single-antigen detection, particularly in low-titre samples. Its single-cartridge format makes it well suited for seroepidemiological surveys and diagnostic use, including in resource-limited settings where rapid and reliable antibody detection is essential. Although the assay is currently not available from the manufacturer (Coris Bioconcept), our findings provide important evidence on the performance of dual-antigen formats and remain relevant for future assay development and validation in similar contexts.

## Data Availability

The datasets used and/or analysed during the current study are available on reasonable request.

## Abbreviations

BAU: Binding Antibody Units
COVID-19: Coronavirus Disease 2019
ELISA: Enzyme-Linked Immunosorbent Assay
IgG: Immunoglobulin G
LOD: Limit of Detection
NP: Nucleoprotein
RBD: Receptor Binding Domain
SARS CoV-2: Severe Acute Respiratory Syndrome Coronavirus 2
