# Correction: Clinical performance of a dual-target SARS CoV-2 antibody assay using sera from Ghana

**DOI:** 10.1186/s12879-026-13734-4

**Published:** 2026-06-17

**Authors:** Prince Jonathan Pappoe-Ashong, Julius Abraham A. Mingle, Derrick Tetteh, Martha S. Dsane-Lamptey, Joseph A. Oliver-Commey, Peter Puplampu, Christian Jassoy

**Affiliations:** 1 Institute of Medical Microbiology and Virology, University Clinics and Medical Faculty, University of Leipzig, Leipzig, Germany; 2 Department of Medical Microbiology, University of Ghana Medical School, Accra, Ghana; 3 Department of Medicine, University of Ghana Medical School, Accra, Ghana; 4 Ghana Infectious Disease Centre, Ghana Health Service, Accra, Ghana; 5 Ga East Municipal Hospital, Ghana Health Service, Accra, Ghana

**Correction: BMC Infectious Diseases (2025) 25:1552**

10.1186/s12879-025-12012-z

In this article, the author’s name was incorrectly written.

Incorrect: Peter Puplumpu

Correct: Peter Puplampu

The original article has been corrected.

